# Implementing and Disseminating an Evidence-Based Program to Prevent Falls in Older Adults, Texas, 2007-2009

**Published:** 2010-10-15

**Authors:** Marcia G. Ory, Matthew Lee Smith, Angie Wade, Chelsea Mounce, Ashley Wilson, Reuben Parrish

**Affiliations:** Department of Social and Behavioral Health, Texas A&M Health Science Center, School of Rural Public Health; Texas A&M Health Science Center, College Station, Texas; Texas A&M Health Science Center, College Station, Texas; Texas A&M Health Science Center, College Station, Texas; Texas A&M Health Science Center, College Station, Texas; Texas Falls Prevention Coalition, Austin, Texas

## Abstract

**Introduction:**

Falls are a public health problem for the growing population of older adults. We describe a statewide effort to implement and disseminate A Matter of Balance/Volunteer Lay Leader model, an evidence-based fall-prevention program.

**Methods:**

We analyzed 2 secondary databases: 1) a centralized administrative data set to document implementation processes and structures for delivering the program and 2) a common set of outcome measures for assessing the effect of the program on older Texans. We used multivariate analyses to examine changes on key outcome variables, controlling for major covariates.

**Results:**

From 2007 through 2009, we reached 3,092 older Texas residents. Program capacity was built by certifying 98 master trainers and 402 lay leaders and delivering the program in 227 classes through the Area Agency on Aging network. Immediate outcome results were positive, which indicates a pathway to promote more successful aging: 1) increases in falls efficacy, 2) improvements in overall physical activity levels, and 3) reductions in interference with everyday normal routines.

**Conclusion:**

Widespread dissemination of a program to prevent falls can promote active aging among people who would otherwise be at risk for a downward cycle of health and functionality. Creating partnerships among different delivery sectors is needed for building community infrastructure to enhance the health of older adults.

## Introduction

Falls among seniors are one of the most preventable causes of injuries, disabilities, and loss of independence (1,2). In 2007, there were more than 50,000 falls among Texas residents aged 50 years or older, resulting in more than 12,000 hip fractures and almost $2 billion in total fall-related hospital charges (3). Modifiable fall risks, falls, the fear of falling, and related negative sequelae may be reduced through educational and behavioral interventions.

A Matter of Balance/Volunteer Lay Leader model (AMOB/VLL) is an evidence-based activity program for community-dwelling older adults; it is intended to reduce fear of falling and increase physical activity levels among seniors. AMOB/VLL can be implemented in 2 versions: a 4-week version with classes that meet twice a week or an 8-week version with weekly classes (4). Early sessions focus on diminishing fears of falling and encouraging participants to adopt the mindset that falls are preventable. Later sessions assist participants in changing their environments to reduce fall-related risk factors and teach them exercises to increase strength and balance. Certified master trainers teach lay leaders to deliver the program with fidelity.

Working with the leadership of the Texas Association of Area Agencies on Aging, the Texas Falls Prevention Coalition (www.texasfpc.org/index.php) disseminated AMOB/VLL across partnering Area Agencies on Aging (AAA) service areas. The Texas A&M Health Science Center provided standardized protocols for programs documenting implementation processes (program reach and adoption) and assessing program outcomes.

AMOB/VLL is being distributed nationwide as part of the Administration on Aging Evidence-Based Disease Prevention Grant Program. Our objective was to describe the training and delivery processes though which AMOB/VLL is implemented and disseminated throughout the Texas Association of Area Agencies on Aging. The secondary objective was to examine selected key outcome measures to validate positive findings reported in previous studies.

## Methods

We collected administrative- and participant-level data from classes conducted from September 2007 through September 2009. We recruited participants to the program through local AAAs and other partnering community-based organizations. Institutional review board approval was obtained from Texas A&M University. Participation in this study was voluntary, and participants could withdraw from the study at any time.

### Measures

The evaluation team created a detailed evaluation manual to standardize implementation processes and obtain common data across all participating sites (www.srph.tamhsc.edu/research/texashealthylifestyles/tfpc/forms.html).

We collected de-identified administrative information to assess the program implementation and dissemination processes (program training capacity, delivery site type, and geographic spread) from AAA sites. Program coordinators at each participating AAA site kept administrative records that were requested by the evaluation center monthly. This information was checked for completeness and accuracy by the Texas Falls Prevention Coalition coordinator. We obtained information on program capacity, which we defined as the number of master trainers and lay leaders at each participating AAA site. We tracked trainer attrition (the number of active and inactive master trainers and lay leaders) through reports from program coordinators, who kept up-to-date rosters of people available to teach the classes. Consistent with the national implementation of evidence-based programs supported by the Administration on Aging, we used a standardized form to capture the types of delivery organization for each class. We defined program adoption in terms of the number and types of organizations that delivered classes under the auspices of the Texas Falls Prevention Coalition.

Program coordinators who collected administrative data at each participating AAA site coded the class delivery sites as senior centers, residential facilities, community centers, faith-based organizations, health care organizations, workplaces, or others. We used administrative data to illustrate the spread of AAA site participation over time. This information was mapped for each AAA region across the 254 Texas counties. Using participant residential zip codes, we assessed how many participants were served by each AAA site.

We collected baseline assessment data from participants at the beginning of the first class and postintervention data at the end of the last class (session 8). The self-reported assessment questionnaire was 9 pages and consisted of 28 items. Survey instrument items included Likert-type, yes/no, closed-ended, and open-ended questions. The questionnaire took approximately 15 minutes to complete, or longer for respondents who needed assistance.

Participants voluntarily enrolled in Texas Falls Prevention Coalition-sponsored AMOB/VLL classes in 19 AAA participating regions throughout Texas. We included age, sex, race/ethnicity, education level, income, and number of chronic conditions as participant demographic characteristics. We used participant responses — health status indicator variables collected at baseline and postintervention — as outcome variables for this study. The falls efficacy scale (α = 0.814, composite score of five 4-point Likert-type scale items, scored 1 for "not sure at all" and 4 for "absolutely sure") assessed participants' perceived ability to prevent falls and injuries from falls (5,6). The health interference scale (α = 0.924, composite score of four 5-point Likert-type scale items, scored 1 for "not at all" and 5 for "almost totally") measured the amount that health interfered with their everyday activities (social activities, hobbies, chores, shopping) (7). Physical activity was assessed by a variant of Behavioral Risk Factor Surveillance System survey items to assess the number of days in the previous week the participant was engaged in moderate-intensity physical activity for at least 30 minutes. We used several related quality-of-life measures to assess the number of days in the previous 30 that health was reported to be "not good," and the number of days in the previous 30 that the participant was kept from participating in usual activities.

### Data analysis

We examined participant data, collected at the beginning and end of the intervention, by using descriptive and multivariate analyses. Not all participants enrolled in the intervention completed instruments at baseline because not all sites collected data for every class they delivered. For participants with available data, we calculated frequencies of demographic characteristics to describe the reach and participant representativeness. We then performed analyses to identify any systematic biases resulting from missing data. The Pearson χ^2^ and *F* tests were used to test for substantial differences in the percentages or means of selected demographic characteristics for participants who completed baseline and postintervention assessments and those with no postintervention assessments.

For multivariate analyses, we used only participant records with complete baseline and postintervention data on all variables. To analyze the AMOB/VLL data for differences from baseline to postintervention, we used a mixed model that accounted for cluster effects with repeated measures. We controlled for age, sex, race/ethnicity, and general health status in each multivariate model. We performed all analyses in SAS version 9.2 (SAS Institute, Inc, Cary, North Carolina).

## Results

As of October 1, 2009, a total of 3,092 unique participants were recruited throughout Texas. These participants averaged 77 years of age (15% were aged ≥85); most were women (83%) and were high school graduates (82%). A high proportion of disadvantaged seniors enrolled in the programs (30% were from a racial/ethnic minority group and 40% had incomes ≤$15,000/y). Of the 3,092 participants, 87% had baseline data, 56% had postintervention data, and 51% had both.

Before assessing program effects, we conducted a bivariate analysis to examine the potential existence of significant differences between those participants who had baseline data only versus those with both baseline and postintervention data. A few differences emerged. More participants who had complete data at both time periods, and thus were included in the multivariate analysis, were non-Hispanic white (73% vs 64%), had attended college (58% vs 50%), and reported fewer unhealthy days (4.8 vs 5.9).

The Texas Association of Area Agencies on Aging sponsored 4 centralized master trainings. All participating sites were encouraged to send people in their AAA region to become certified, making them eligible to train lay leaders at their local site. As a large state with a commitment to preventing falls for seniors, Texas now has more trainers than any other state delivering AMOB/VLL. Of the 98 people trained as master trainers, 83 were still actively training. Of the 402 people trained to be lay leaders, 278 were still active. Given these data, the Texas Falls Prevention Coalition leaders recognized lay leader attrition as a problem. Local AAA sites now give more attention to recruitment and retention planning; their goal is to achieve higher retention of volunteer lay leaders and provide support services more efficiently.

As of October 1, 2009, 227 AMOB/VLL classes had been delivered at 146 unique sites. The most frequent implementation sites were senior centers (77 classes) and residential facilities (63 classes). Other sites included faith-based organizations (23 classes), health care organizations (12 classes), and workplaces (7 classes). Programs retained most participants: 76% of class participants completed at least 5 of 8 sessions. The average class size was 15 participants, which was larger than the ideal class size of 8 to 12 participants.

Twenty-six of the 28 AAAs contracted with the Texas Association of Area Agencies on Aging to deliver the AMOB/VLL program, for a potential reach of 236 of 254 Texas counties (Figure). Each participating AAA agreed to hold a minimum of 6 classes, resulting in approximately 100 participants each. Through this infrastructure, AAAs conducted 227 classes in the 2-year timeframe or an average of nearly 9 classes for the participating AAAs. However, we noted substantial variation in the number of classes delivered; the highest-yield AAA site conducted 31 classes, and 5 sites offered no classes. Although the intent was to expand the program statewide, we found a clustering of programs in more populated areas of the state and limited penetration in the least populated areas.

**Figure 1:**
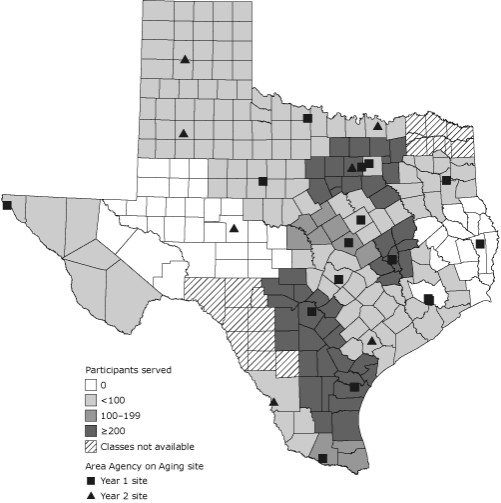
Geographic reach of A Matter of Balance/Volunteer Lay Leader model in Texas. This map illustrates the sequential uptake of Area Agencies on Aging in the delivery of A Matter of Balance/Volunteer Lay Leader model during the 2 years (2007-2009) of this study. Area Agency on Aging regions are shaded on the basis of the number of participants they served as of October 1, 2009.

**Table:** 

For the delivery of A Matter of Balance/Volunteer Lay Leader model at Area Agency on Aging (AAA) sites, the number of participants in each region were 219 for Alamo, 248 for Bexar County, 205 for Brazos Valley, 98 for Capital, 163 for Central Texas, 323 for Coastal Bend, 0 for Concho Valley, 62 for Dallas County, 0 for Deep East Texas, 7 for East Texas, 33 for Golden Crescent, 0 for Harris County, 95 for Heart of Texas, 1 for Houston-Galveston, 158 for Lower Rio Grande Valley, 296 for North Central Texas, 35 for North Texas, 21 for Panhandle, 0 for Permian Basin, 29 for Rio Grande, 0 for South East Texas, 8 for South Plains, 3 for South Texas, 91 for TEXOMA, 205 for Tarrant County, and 48 for West Central Texas.
For year 1, sites existed in most AAA regions. In addition to those sites, the Pan Handle, Concho Valley, Golden Crescent, South Plains, South Texas, Tarrant County, and TEXOMA regions were added in year 2. Classes were not offered in ARK-TEX or Middle Rio Grande regions.

Results were uniformly positive for AMOB/VLL participants (Table). Adjusted for key covariates (age, sex, race/ethnicity, self-assessed health), these multivariate analyses show strong effects of the intervention on falls efficacy. Other outcome variables showed more modest effects, including number of days physically active and reductions in health interference. An effect was found for physically unhealthy days but not for mentally unhealthy days.

## Discussion

Our findings demonstrate the training and delivery structures necessary for the widespread dissemination of evidence-based programs. Not only do programs need to be *manualized* so others may easily adopt them (8), training capacity must also be adequate to meet the increased delivery demand with fidelity (9). Although state funding provided to Texas Association of Area Agencies on Aging made the infrastructure for disseminating AMOB/VLL statewide possible, programs were not established in all participating counties as anticipated. In the dissemination of any innovation, however, there will be early and late adopters (10). Moreover, geographic spread may not occur evenly in a particular AAA region since the AAAs serve multiple counties in a region, and delivery may be concentrated in a limited service area in a total AAA coverage area.

Additional investigation is needed to more systematically understand why some AAAs were more successful than others in implementing the program. Consistent with prior findings (11), organization size seems to be a factor: AAA sites that had more infrastructure resources could expend extra effort to recruit delivery sites and participants.

Our findings regarding participant outcomes were consistent with those of the original randomized clinical trial (5) and the initial translational research study (6). This research confirms that evidence-based fall-prevention programs are a pathway to healthier aging by modifying risk factors for falls that are associated with a downward cycle of fear and inactivity (2). Of special note, this research examined a broader range of outcomes than employed by the Maine program developers. Our investigation provides an opportunity to advance knowledge about the influence of a low-intensity fall-prevention program on reducing interference in activities of daily life and unhealthy days.

Although the number of participants in this study is larger than that of other examinations of AMOB/VLL (6), there was a substantial decrease from those enrolled to those with complete baseline and postintervention assessments. At the first level (from enrollment to baseline assessment), we believe this decrease reflected the ability of individual delivery sites to collect data instead of indicating any specific systematic bias for an individual participant. However, without data on all participants, we cannot determine whether those who did not become part of the database differed. We described the ways that participants with only baseline data differed from those with full data, and as in many intervention studies, more participants with more complete data were non-Hispanic white, healthier, and more educated (12). To help minimize the effects of these differences, these key covariates were used as controls when examining differences between preassessment and postassessment scores. The decrease in participation from baseline to postintervention assessment is typical in community-based studies.

Although we went beyond limited data-collection efforts in other states that implemented AMOB/VLL, we did not include direct association of intervention benefits with fall reduction, objective physical functioning measures, or links to health care use and costs that can make a stronger case for reimbursement (eg, health insurance payer reimbursement by public or private insurance mechanisms). Discussion is taking place at the national level of the need to document programmatic costs and compare these costs with reported outcomes. This is not possible in the current study, where analyses were conducted only at the immediate postintervention period. This study is also subject to a common research limitation — the lack of long-term follow-up data (13). Some sites are collecting 6-month follow-up data that can begin to address the long-term effects of these community programs; however, these data are not currently available. Similarly, data should be collected on factors associated with program maintenance at the organizational level. We note some sites were active in the first year but not the second year. A framework exists for understanding the sustainability of community health promotion programs (11), and future research should focus on understanding the direction of community programming on local, regional, and state levels.

The recent movement toward building healthy communities (14) may guide interventions intended to promote active aging. In this study, the most prevalent delivery sites were those where older adults live (residential facilities such as senior housing, retirement communities, or assisted-living facilities) or those associated with organizations already serving seniors (such as senior centers).

Most local AAAs have reached out to nontraditional aging partners for program delivery (such as parks and recreation departments or general community centers), and these types of partnerships are needed to broadly disseminate the intervention. We recommend that these types of evidence-based programs be implemented where seniors live, play, or pray, to achieve healthy aging and healthier communities (15). A broader view of falls prevention best practices is needed that will go beyond evidence-based behavioral programming to appreciate the active and supportive roles of surrounding communities.

## Figures and Tables

**Table:** Effectiveness of A Matter of Balance/Volunteer Lay Leader Fall-Prevention Program, Texas, 2007-2009

**Variable^a^**	Baseline	Postintervention	n	*t* Value	*P* Value	Cohen *d*
Falls efficacy scale^b^	12.5	14.1	1,221	19.97	<.001	1.14
No. of days physically active^c^	3.2	3.5	1,233	4.77	<.001	0.27
No. of unhealthy physical days^c^	2.7	2.0	1,267	2.50	.01	0.14
No. of unhealthy mental days^c^	1.6	1.4	1,280	1.16	.25	0.06
No. of days kept from usual activity^c^	1.5	0.9	1,296	3.00	.003	0.17
Health interference scale^d^	8.0	7.5	1,245	4.28	<.001	0.24

a Covariates were age, sex, race/ethnicity, and general health status. Analyses accounted for cluster effects (by class).

b Assessed perceived ability to prevent falls and injuries from falls by using the composite score of five 4-point Likert-type scale items, ranging from 5 to 20, scored 1 for "not sure at all" and 4 if "absolutely sure."

c Assessed for the previous 30 days.

d Assessed perceived amount that health interfered with everyday activities by using the composite score of four 5-point Likert-type scale items, ranging from 4 to 20, scored 1 for "not at all" and 5 if "almost totally."
